# Technological Methods for Controlling the Elastic-Deformable State in Turning and Grinding Shafts of Low Stiffness

**DOI:** 10.3390/ma15155265

**Published:** 2022-07-29

**Authors:** Antoni Świć, Arkadiusz Gola, Olga Orynycz, Karol Tucki, Jonas Matijošius

**Affiliations:** 1 Department of Production Computerisation and Robotisation, Faculty of Mechanical Engineering, Lublin University of Technology, ul. Nadbystrzycka 36, 20-618 Lublin, Poland; a.swic@pollub.pl; 2 Department of Production Management, Faculty of Engineering Management, Bialystok University of Technology, ul. Wiejska 45A, 15-351 Bialystok, Poland; o.orynycz@pb.edu.pl; 3 Department of Production Engineering, Institute of Mechanical Engineering, Warsaw University of Life Sciences, ul. Nowoursynowska 164, 02-787 Warsaw, Poland; karol_tucki@sggw.edu.pl; 4 Institute of Mechanical Science, Vilnius Gediminas Technical University, J. Basanavičiaus str. 28, LT-03224 Vilnius, Lithuania; jonas.matijosius@vilniustech.lt

**Keywords:** low stiffness shaft, control, elastic-deformable state, technological methods of shaft processing, turning, grinding, processing quality

## Abstract

The article presents original technological methods that allow the improvement of the accuracy of the turning and grinding of elastic-deformable shafts by increasing their stiffness by controlling the state of elastic deformations. In particular, the adaptive control algorithm of the machining process that allows the elimination of the influence of the cutting force vibration and compensates for the bending vibrations is proposed. Moreover, a novel technological system, equipped with the mechanism enabling the regulation of the stiffness and dedicated software, is presented. The conducted experimental studies of the proposed methods show that, in comparison with the passive compliance equalization, the linearization control ensures a two-fold increase in the shape accuracy. Compared to the uncontrolled grinding process of shafts with low stiffness, the programmable compliance control increases the accuracy of the shape by four times. A further increase in the accuracy of the shape while automating the processes of abrasive machining is associated with the proposed adaptive control algorithm. Moreover, the initial experiments with the adaptive devices prove that it is possible to reduce the longitudinal shape inaccuracy even by seven times.

## 1. Introduction

The analysis of the literature shows that about half of all machine parts are rotating parts: shafts, discs, sleeves, cylinders, etc. [1,2]. It is estimated that 12% of all the parts used in industries are shafts of a low stiffness [3,4]. They are used in the aviation industry (resilient, flexible, and torsion shafts, springs, and screws) [5], the tool industry (various types of instruments, mechanisms, precision and special tools, drills, reamers, taps, and boring bars) [6], machine industry (shafts, turbine and pump rotors, and lead screws) [7,8], in agricultural machinery (tractor and combine shafts) [9], the automotive industry [10], elements of photovoltaic installations [11], and others.

Parts with low stiffness are characterized by disproportionate dimensions and low stiffness in specific sections and directions [12]. High requirements are also placed on the parameters of the geometric shape, mutual position of the surfaces, linear dimensions, and surface quality [13,14].

The specificity of low-rigidity shafts’ processing makes it difficult to obtain certain parameters of the shape accuracy, dimensions, and surface quality [15,16]. The low stiffness of the shaft, as compared to the rigid assemblies of the machine tool, causes vibrations under certain conditions [17]. During machining, there are many factors that disturb and destabilize this process (the deformation of the parts, tools, devices, chips, dust, etc.), which affects the deterioration of the processing conditions for shafts with low stiffness [18,19]. Traditional methods of achieving the accuracy of machining shafts with low rigidity, using multi-pass machining, reduced the machining parameters, steadies, and additional steps and manual lapping, lead to a significant reduction in efficiency, do not meet the modern requirements of automation, and are uneconomical and inefficient [20].

The quality of the machining of the parts on metal cutting machines can be significantly increased as a result of the application of the developed technological machining methods, enabling the adjustment of the susceptibility of the technological system and appropriate control systems of the machine’s tools and parameters of the technological system [21]. It is especially important for machining parts with low stiffness when it is necessary to ensure their appropriate stiffness to enable efficient machining [22]. The accuracy and quality of machining may be increased as a result of the development and application of appropriate (close to optimal) structures of the control systems [23,24].

The problem of controlling turning and grinding processes has been discussed by numerous authors. Among the solutions proposed in the literature, the most frequently adopted one is the model of interferences acting on the turning process as consisting of two components: a component representing an approximately linear trend and a random component with a wide frequency band [25]. The first component is mainly related to the temperature deformations of the machine tool and wear of the cutting edge [26]. These interferences do not change significantly within one machining cycle and can be compensated for by setting the static operating point in each subsequent machining cycle [27]. The high-frequency component is mainly conditioned by changes in the machining allowance, changes in the material hardness, and other complex phenomena occurring during the turning process [28]. The basic high-frequency component compensation method is used to control the elastic deformations of the turning process system [29,30].

Apart from obtaining a high machining accuracy in the turning process, it is important to increase the efficiency of the grinding process, which can provide a considerable economic benefit [31]. Some machine tools are constructed and operated with the aim of achieving the highest possible efficiency, while others are mainly used for precision machining [32]. Such specialization of machine tools requires the development of specific optimization and control criteria suitable for the individual classes of those tools [33,34]. The point of departure for controlling all the classes of machine tools is the stabilization of the cutting forces and the associated elastic deformations [35,36].

The provided analysis of the literature shows that, although there are many publications that present solutions to particular problems with online machining parameters’ measurement and control (see, e.g., [37]), the stability prediction in the straight turning’s (see, e.g., [38]), or just the conventional and intelligent methods for the machining accuracy’s, improvement (see, e.g., [39]). Unfortunately, there is no research devoted to the adaptive controlling of machining processes that allows the elimination of the influence of the cutting force vibration on the machining results and compensate for the bending vibrations during the grinding process.

The goal of this article is to present developed technological methods of turning and grinding low rigidity shafts with controlling the state of elastic deformations by adjusting the susceptibility of the technological system.

## 2. Technological Methods of Machining Shafts with Low Stiffness

### 2.1. The System of Adjustment of the Compliance of the Technological System

On the basis of the conducted theoretical and experimental research, the technological methods of machining by turning and grinding with control of the state of the elastic deformation of shafts with low stiffness in the technological system were developed. The developed methods are focused on adjusting the susceptibility of the technological system and continuous adjustment of the process of machining by putting bending moments to the ends of the machined part.

**Figure 1:**
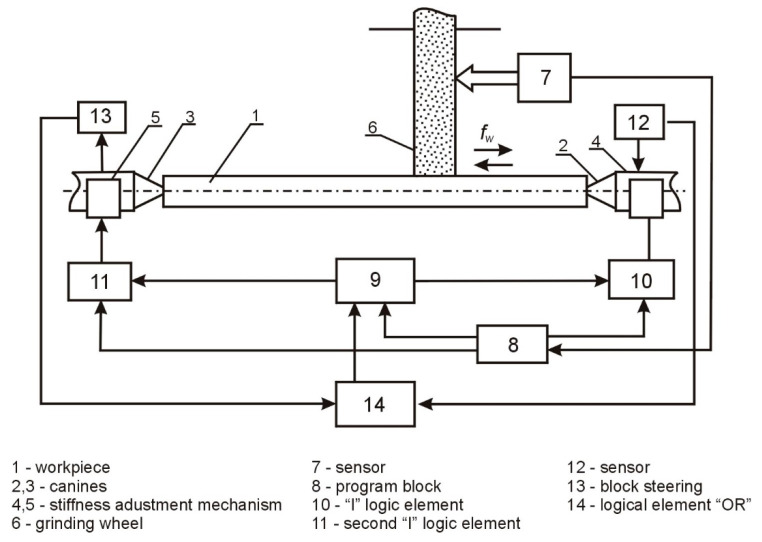
Diagram of the adjustment system of the technological system’s compliance during grinding.

The adjustment of the compliance of the technological system was carried out by means of a specially designed system of adjustment of the compliance of the technological system during processing. The block diagram of the control of the compliance of the technological system during grinding is presented in Figure 1.

In the beginning, the control block, 9, is turned on (Figure 1), and the voltage is applied to the stiffness adjustment mechanisms, 4 and 5, located in centers 2 and 3, and then rotates part 1 and the grinding wheel, 6; the grinding is started with a longitudinal feed, FP. Based on the signals from the sensor, 7, determining the location of the grinding wheel along the length of the machined surface, the voltage pulses are generated in the program block, 8, which flow to the control block, 9, and to one of the inputs of the logic element “I”-10. This element connects the mechanism adjusting the stiffness, 4, with the control block, 9, and changes the stiffness of the tailstock, 2, according to the program. From the sensor, 12, a signal carrying information about the value of the stiffness of the tooth, 3, flows via the logic “OR”-14 to the control block, 9, and provides feedback to increase the speed of the system. As the wheel, 6, continues to be moved along the work surface by the signals of the program block, 8, flowing through the second logic element “I”-11, the second part of the device for adjusting the stiffness of the wheel, 3, is activated, according to the principle set out above. After finishing the machining, the control block, 9, is turned off, and the grinding wheel, 6, moves away from the shaft.

As the wheel position sensor, a differential electromagnetic linear displacement transducer with helical surfaces of magnetic drives is used, in which the electric windings are arranged bifilarly. The diagram of switching on the converter can be presented as a sensitive medium-sized current amplifier. The diagram transforms the variable voltage of the secondary windings of the “OR” converter into a constant voltage, the polarity of which is determined by the direction and the average value of the rotor displacement from the zero position.

In the control system of the compliance of the technological system, specially designed tailstocks are used, equipped with mechanisms enabling the regulation of the stiffness and software devices controlling the machining.

**Figure 2:**
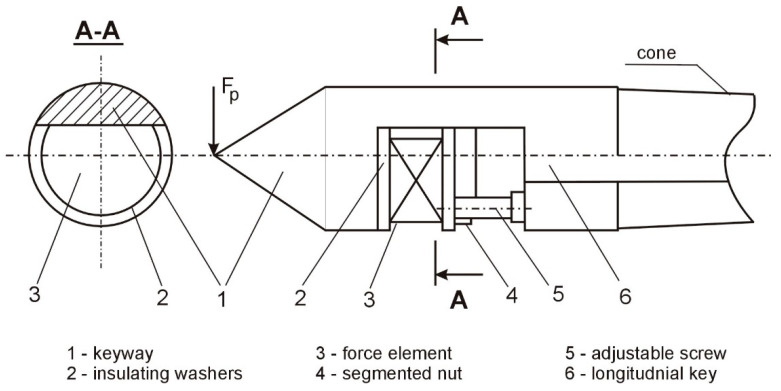
The structure of the claw with adjustable stiffness.

**Figure 3:**
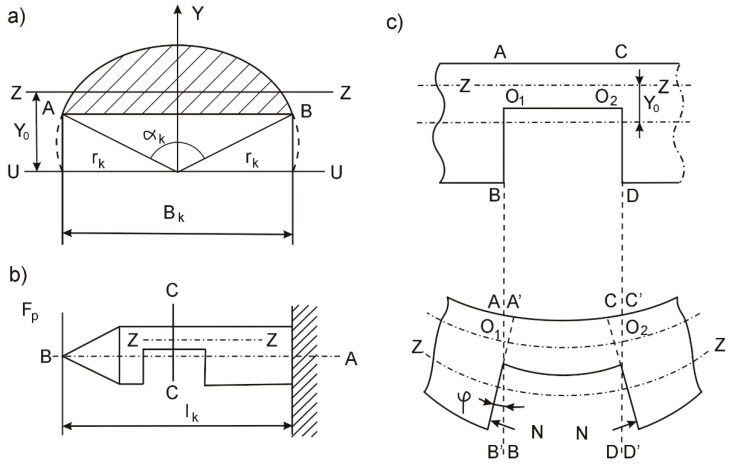
The tailstocks that allow the adjustment of the stiffness: (**a**) shape of the cross-section, (**b**) calculation scheme of the tailstock load, and (**c**) the nature of the deformation of the weakened tailstock.

The structure of the developed mechanism and the shape of the weakened transverse section of the tusk are shown in Figure 2 and Figure 3.

In the stiffness regulation mechanisms, standard tusks were used, in which a one-sided longitudinal through-key was made perpendicular to the axis. In these grooves, there are discs made of piezoceramic layers with a diameter of 25 mm and a thickness of 1 mm. Between the dials, there are layers with a silver-plated brass foil. The set of targets is joined with BF-2 glue and dried; thus, the electrically conducting parts are insulated with a dense mixture of epoxy resins. The mechanism realizes a force of up to 60 N/cm^2^.

In the groove of the claw, 1 (Figure 2), between the insulating washers, 2, a force element, 3, consisting of piezoceramic layers, is placed. The element, 3, is fastened in the groove by means of a segmented nut, 4, and an adjustable screw, 5. A longitudinal groove, 6, is made to lead the cables from the element to the control scheme along the spine. Such a design provides:

- a convenient place for embedding and distributing the force elements;
- a reduction of the initial compliance of the tailstocks, as measured by the compliance of the workpieces;
- the enhanced stiffness of the centers in the direction of the tangential component of the cutting force;
- a wide range of changes in the stiffness of the tailstocks.

Calculations of the initial susceptibility of the weakened tailstocks were performed using the known relationships of the strength of the materials; therefore, the tooth set in the headstock or the tailstock of the machine tool is considered to be a beam of length, *l_k_*, restrained by the end, *A*, and loaded with a force, *F_P_*, in the end, *B* (Figure 3b).

The beam end’s deflection is determined using the equation:

(1) $$Y_{B}=\frac{F_{P}l_{k}^{3}}{3EI_{k}},$$

where *F_p_* is the radial cutting force, *E* = 2 × 10^11^ [Pa] denotes the tensile modulus, *l_k_* is the length of the support part of the tailstock, *I_k_* is the section moment of the inertia, *C-C*, m^4^.

Taking into account that the susceptibility is *ω* = *Y/F_p_* after the transformation, the relationship for calculating the tailstock susceptibility takes the form of:

(2) $$\omega =\frac{0,6\;10^{-12}l_{k}^{3}}{I^{k}},$$

where ω is the susceptibility in [mN].

For the weakened *C*-*C* cross-section, assumed in Figure 3a, the moments of inertia, *I_Y_*, *I_Z_*, and other parameters, are determined from the relationship:

(3) $$B_{k}=2r_{k}\sin\frac{\alpha_{k}}{2},$$

where *r_k_* is the tailstock radius in the cross-section, and $\alpha_{k}$ is the arc of the contact along the length of the key (determining its cross-sectional area).

- The cross-sectional area (*S*):

(4) $$S=\frac{r_{k}^{2}}{2}(\alpha_{rad}-\sin\alpha_{k}),$$

- The distance from the symmetry axis of the *U-U* tailstock to the neutral *Z-Z* axis of the bending section (*y*_0_):

(5) $$y_{0}=\frac{B_{k}^{3}}{12S_{}}=\frac{4r_{k}\sin^{3}\alpha_{k}}{3\alpha_{rad}-\sin\alpha_{k}},$$

where *B_k_* is the keyway’s length in the tailstock.

- The moments of inertia of the cross-section:
  - with regard to the *U-U* axis of the tailstock’s symmetry (in the direction of the force):
    (6) $$I_{k}=\frac{r_{k}^{4}}{8}(\alpha_{rad}-\sin\alpha_{k}\cos\alpha_{k}),$$
  - relative to the central *Z*-*Z* axis of the bending section (in the direction of the *F_P_* force):
    (7) $$I_{Y}=I_{k}-S_{}y_{0}^{2}$$
  - with respect to the symmetry axis of the cross-section, *O_Y_*, (in the direction of the component force, *F_c_*):
    (8) $$I_{Z}=\frac{r_{k}^{4}}{8}[\alpha_{rad}-(\sin\alpha_{k}-\frac{2}{3}\sin\alpha_{k}\sin^{2}\frac{\alpha_{k}}{2})]$$

Based on the above dependencies, the calculations were made on the susceptibility of the weakened tailstocks with a different cross-sectional area and determined by the size of the angle, α*_k_*, and the specific elastic deformations resulting from the action of the cutting force components. Standard tusks, with a cylindrical part diameter of 31 mm and 22 mm, and the length of the cantilever part, with a position in the headstock and tailstock, where *l_k_* = 70 mm, were used as blanks. The calculation results are presented in Table 1.

### 2.2. Adaptive Control of the Machining Process

**Figure 4:**
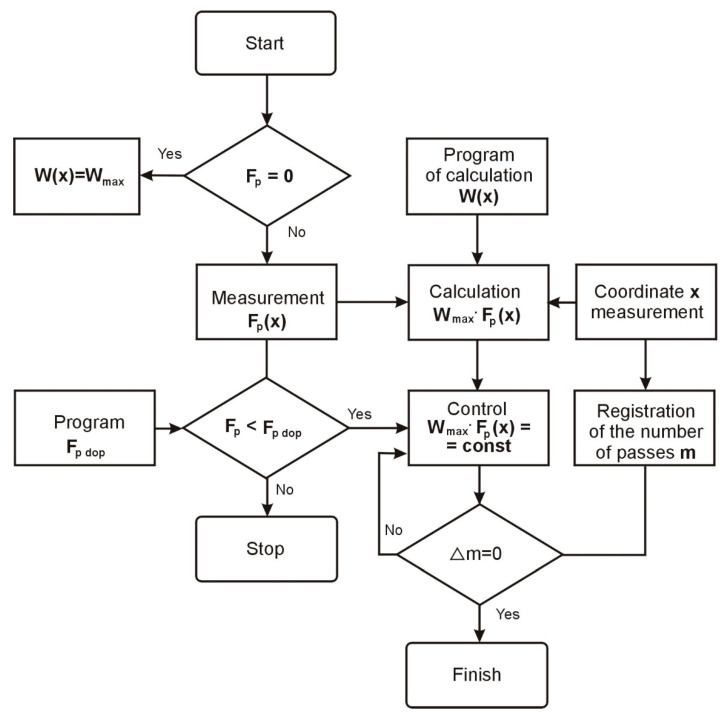
Block diagram of the adaptive control algorithm of the grinding process.

In order to eliminate the influence of the cutting force vibration on the machining results, an algorithm of adaptive control of machining processes was developed, the block diagram of which is presented in Figure 4. For the practical implementation of the algorithm, the software control system of the technological system is additionally equipped with counters for the number of passes and calculation blocks based on a microprocessor.

**Figure 5:**
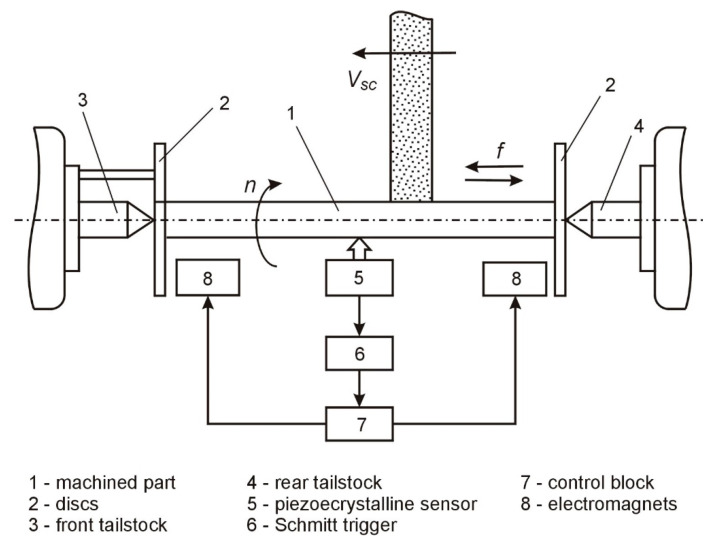
Block diagram of measurement and compensation of bending vibrations during grinding.

Theoretical research shows the possibility of inducing low rigidity bending vibrations in the grinding process, commensurated under certain conditions with the magnitude of the static bending deformations. To measure the bending vibrations occurring in the grinding process in order to compensate for their influence on the machining accuracy, a special system was developed, the functional block diagram of which is shown in Figure 5.

The system works as follows (Figure 5): Part 1, at the ends of which the disks, 2, are rigidly fixed, is placed in the front, 3, and rear, 4, centers of the roller grinder. The machining process takes place with the rotation of the parts at the rotational speed, *n*, the grinding wheel at the speed *v_sc_,* and with the transverse feed, *f.*

In this case, as a result of the action of various disturbances (the instability of the cutting forces, variable stiffness of the elastic system, wheel imbalance, etc.), the bending vibrations of the parts are induced, which are measured by the piezocrystalline sensor, 5. For the vibration compensation, the signal from the sensor, 5, is proportional to the vibration frequency; through the Schmitt projector, 6 goes to the control block, 7.

## 3. Experimental Research in the Field of Increasing the Accuracy of the Machining Process

Experimental studies of the methods of increasing the accuracy of machining during grinding, as a result of adjusting the compliance of the technological system, were carried out under the following conditions:

- The stiffness of the tusks is much higher than the stiffness of the blank (the compliance is not evened out and does not adjust);
- The compliance of the tusks is equal to the double compliance of the blank and does not change during the processing (the passive compensation of the compliance);
- The compliance of the tailstocks is adjusted along the treated surface (the active compliance compensation).

The samples were semi-finished products of low stiffness with diameters *d* = 8 mm and *d* = 14 mm, for which the ratio of length to diameter, *L/d*, is 20. The tests were carried out with the following grinding conditions: the transverse feed (*f_p_*)—0.01 mm/double pass, longitudinal feed (*f_w_*), 16 mm/rev, cutting speed, 12 m/s.

The grinding force was calculated depending on the dimensions of the samples, the grinding parameters adopted in the tests, and the coordinates of the grinding wheel’s position in relation to the machined surface. The average value of the calculated perpendicular stabilized force is *F_p_* = 15 N. For the stiffness adjustment mechanisms, standard tailstocks (*d* = 22 mm) were used, in which a one-sided key is made perpendicular to the longitudinal axis.

A set of round ceramic plates was placed in the inlets. This mechanism allows for obtaining pressures with values up to 60 N/cm^2^.

The grinding of each sample was performed with 10 double passes. The shape deviation was determined from measurements at five sections and at the ends of the shaft. The absolute value of the shape deviation was taken to be half the difference between the largest and smallest diameters along the machining length. The smallest dimension was taken as the zero deviation.

Standardized tailstocks with a diameter of 22 mm were used in the tests. The compliance was compensated by reducing the cross-section of the standard centers with a special groove. The dimensions of this cross-section may vary depending on the dimensions and compliance of the test samples. The obtained compliance was 4 µm/N with an extension of the tailstocks of 85 mm and 2 µm/N with an extension of the tailstocks equal to 70 mm.

**Figure 6:**
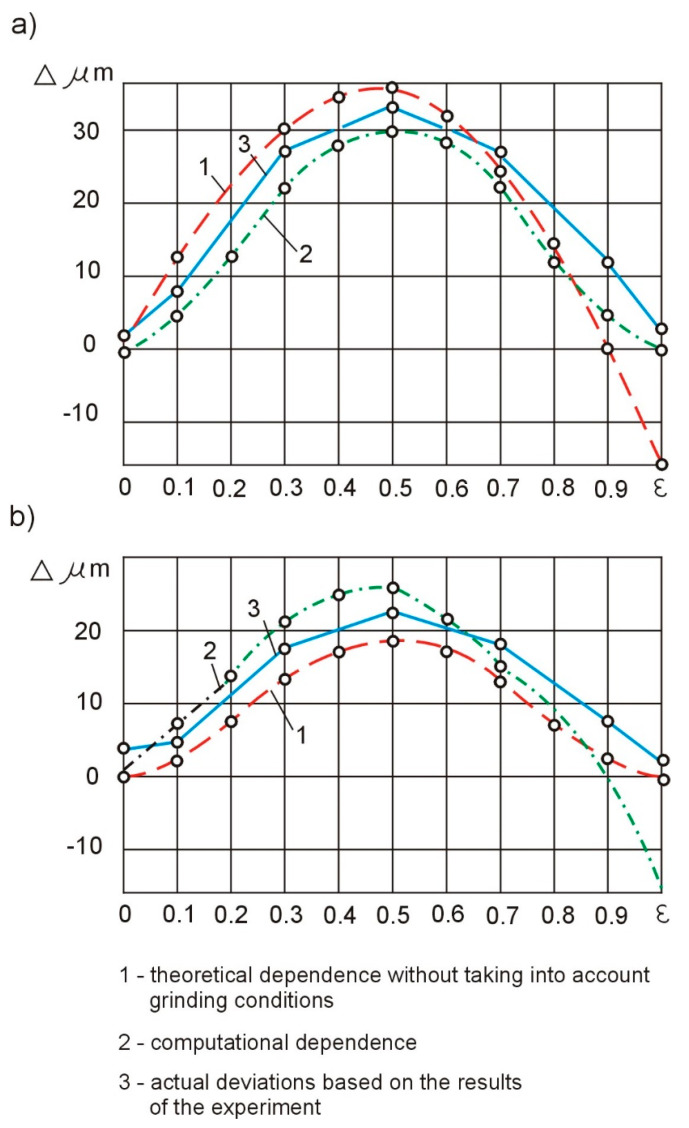
Compliance of the technological system with grinding: (**a**) errors of cylindricity of shafts mounted in rigid centers for diameter *d* = 8 mm, and (**b**) errors of cylindricity of shafts mounted in rigid centers for diameter *d* = 14 mm.

The results of the experimental tests of the samples with diameters of *d* = 8 mm and *d* = 14 mm are presented in a graphical form in Figure 6a,c. The theoretical curves, 1, obtained analytically, show the deviation of the shape (Δ) in the case when the stiffness of the centers significantly exceeded the stiffness of the blank. The compliance of the technological system is not evened out, and the influence of the cutting parameters on the accuracy of the machining was not taken into account. Curves 2, obtained in an analytical manner, characterize the deviation of the shape of the tested samples, taking into account their dimensions and the cutting parameters adopted for the tests. The actual deviation of the shape of the experimental samples (the mean value for six tests) using the rigid tusks is presented with the help of three curves.

By analyzing curve 2, one can notice the difference in the shape deviations on the centers (*ε* = 0 and *ε* = 1), which is explained by the geometric inaccuracy (a shift of the neutral axis to the grinding wheel) or the greater stiffness of the headstock of the machine tool on which the tests were carried out. The analysis shows that for the machine tool used, the geometric inaccuracy is 16 μm and does not depend on the dimensions of the samples and the grinding conditions. Compensation for this inaccuracy can be achieved by increasing the stiffness and, consequently, reducing the tailstock deflection. For the assumed value of the grinding resistance force, *F_p_* = 15 N, the initial susceptibility should be reduced by about 1 µm/N. As a result of the reduction of the tailstock deflections at the right end of the part, the depth of the grinding wheel is cut with each pass increased, so the total amount of the allowance was removed during the machining.

**Figure 7:**
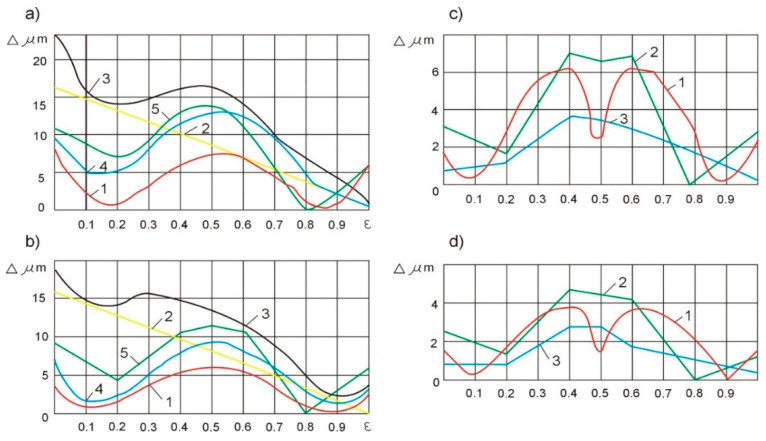
Shaft cylindricity errors: when adjusting the susceptibility of the technological systems: (**a**) for *d* = 8 mm and (**b**) for *d* = 14 mm; when adjusting the susceptibility of the technological systems: (**c**) for *d* = 8 mm and (**d**) for *d* = 14 mm.

In the second case, the susceptibility of the technological system was compensated by deliberately weakening the tailstocks. In the absence of the geometric inaccuracy of the machine tool, the flexibility of the headstock and the tailstock should be the same and amount to samples with diameters of *d* = 8 mm and *d* = 14 mm, respectively (4 μm/N and 2 μm/N). The theoretical deviation of the shape when adjusting the compliance is shown by curves 1 in Figure 7a,b.

A specific geometric inaccuracy of the machine tool (curve 2) introduces a systematic inaccuracy to the machining result. The theoretical deviation of the shape, in this case, is represented by curves 3 (Figure 7a,b). The greatest deviation of the shape is 23 mm for shafts with a diameter of *d* = 8 mm and 19 mm for shafts with a diameter of *d* = 14 mm. These values are close to the experimental shape deviation values for rigid tailstocks—32 µm and 22 µm, respectively. It follows that with the occurrence of geometrical inaccuracies of the machine tool, even the equalization of the compliance of the technological system does not ensure an increase in the accuracy of the shape.

In order to compensate for the initial shift of the part axis caused by the geometric inaccuracies of the machine tool, the compliance of the headstock and the tailstock during the experimental tests were different. For the samples with a diameter of *d* = 8 mm, the compliance of the headstock was assumed to be 4 µm/N and the tailstock 3 µm/N. For the samples with diameters of *d* = 14 mm, the compliance of the headstock was assumed to be 2 µm/N and the tailstock 1 µm/N. With these values of compliance and with the occurrence of geometric inaccuracies, the theoretical deviation of the shape of the samples is presented in Figure 7a,b, showing the geometric inaccuracy of the machine tool and increased machining accuracy.

The results of the experimental tests are presented in Figure 7a,b by means of curves 5, which indicate the correctness of the above assumptions. With the so determined, taking into account the compensation, geometric inaccuracy of the headstock and tailstock, and change depending on the shaft length, the greatest experimental shape deviation obtained is equal to 14.5 µm for the shafts with a diameter of *d* = 8 mm and 11.5 µm for wa, which leads with a diameter of *d* = 14 mm. The shape deviation values are almost two times smaller than with the uniform weakening of both tailstocks.

When adjusting the susceptibility of the technological system along the machining length, experimental tests were carried out for two cases:

(a) the linearization regulation and
(b) program regulation, according to the equation:

(9) $$\omega_{5}(\varepsilon )=1-8\varepsilon^{2}{\left(1-\varepsilon \right)}^{2}$$

where *ω* is the system susceptibility, and *ε* is the relative susceptibility.

**Figure 8:**
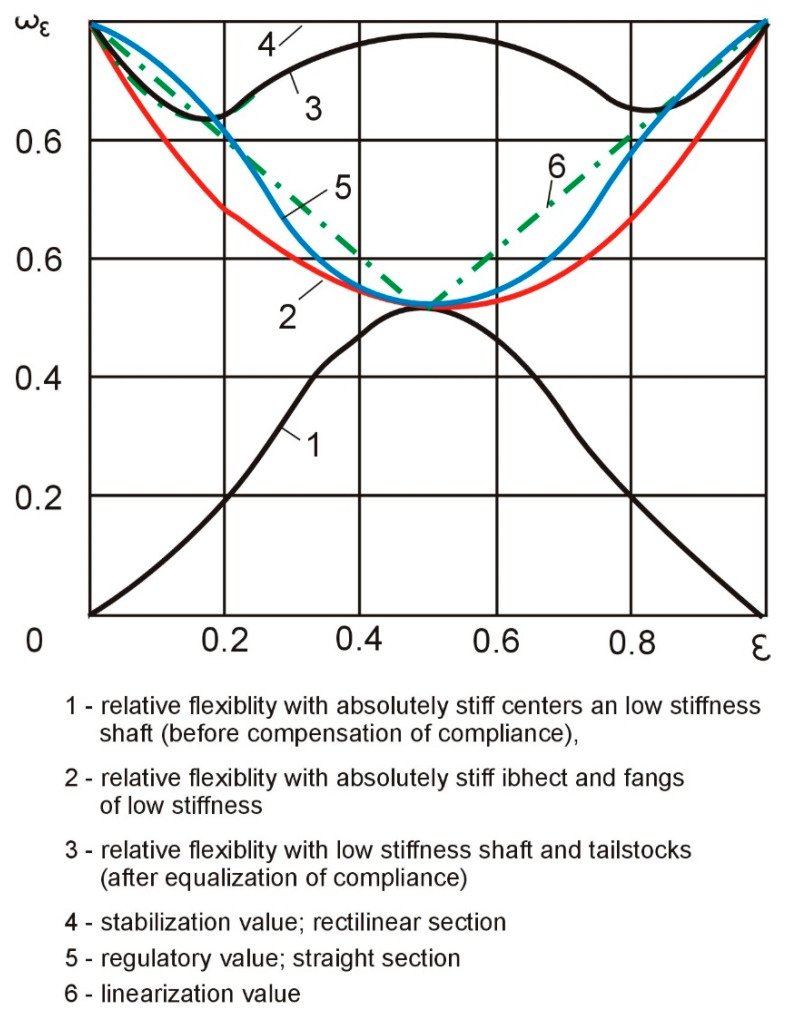
The relative flexibility of the elastic technological systems, depending on the length of machining.

In the case of the linearization control of the technological system’s compliance along the machining length, curve 5 (Figure 8) is replaced by the linearized curve 6 (Figure 8), consisting of two line sections with an inflection at point *ε* = 0.5.

For the samples with a diameter of *d* = 8 mm, the compliance of the technological system was obtained with a value of 3 μm/N for *ε* = 0; then, with a linear displacement of the grinding wheel along the blank, the compliance value was reduced to 2 μm/N at *ε* = 0.5 and again increases to 4 µm/N with *ε* = 1.0. Similarly, for the samples with a diameter of *d* = 14 mm, the compliance decreases from 1.5 µm/N with *ε* = 0 to 1.0 µm/N with *ε* = 0.5, and then increases to 2 µm/N with *ε* = 1.0.

The theoretical values of the deviations of the shape of the samples with diameters *d* = 8 mm and *d* = 14 mm with a linearized regulation are presented using curves 1, and the results of the experimental tests using curves 2 (Figure 7c,d).

In the case of the software control of the compliance of the technological system in accordance with the relation (9), the theoretical control curve takes the form of curve 5 (Figure 8). Such an adjustment should theoretically result in a zero shape deviation. However, the instability of the grinding process and the susceptibility of the technological system along the length of the machining, as well as the errors in the devices implementing the control program, cause actual deviations in the shape of the samples, presented by curves 3 in Figure 7c,d.

The analysis of the results of the experimental tests shows that in comparison with the passive compliance adjustment (Figure 7a,b—curves 5), the linearization control (Figure 7—curves 2) ensures a twofold increase in the shape accuracy and the program control (Figure 7c,d—curves 3) approximately fourfold. Compared to the uncontrolled grinding process of the low stiffness shafts with diameters of *d* = 8 mm and *d* = 14 mm (Figure 7a,b—curves 3), the programmable compliance adjustment increases the shape accuracy by one row. Further increasing the accuracy of the shape with the automation of the processes of abrasive machining of the shafts with low rigidity is associated with the use of adaptive control.

**Figure 9:**
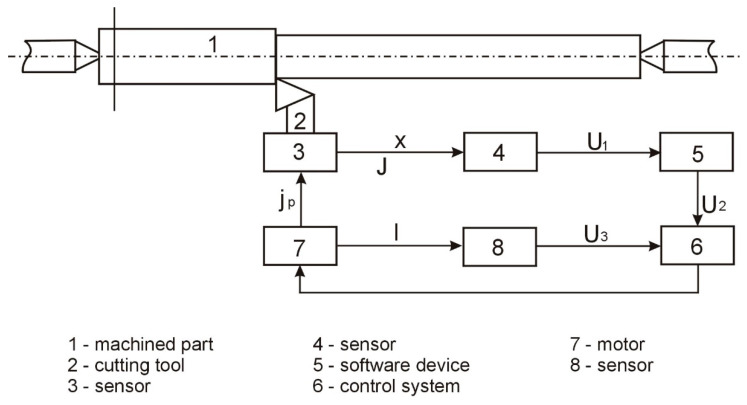
Functional diagram of the adaptive device.

Machining with the adaptive stiffness control was performed using a device, the functional diagram of which is shown in Figure 9. When the tool is displaced, x, the signal, *U*1 *= f* (*x*), from the sensor, 4, is sent to the software device, 5, in which the signal, *U*1 *= f* (*x*), is shaped according to the program-determined change in the stiffness, *j_c_*, of the system and the length, l, of the clamped part of the elastic element. The control system, 6, simultaneously receives signals *U*_2_ and *U*_3_ = *f* (*I_φ_*) from the sensor, 8, which is proportional to the actual length of the clamped part of the elastic element. The divergence signal, U4, is sent to the motor of the mechanism, 7, which moves the movable resistance to the position in which the stiffness of the regulated node becomes equal to the given (for the section of the part processed at a given moment).

**Figure 10:**
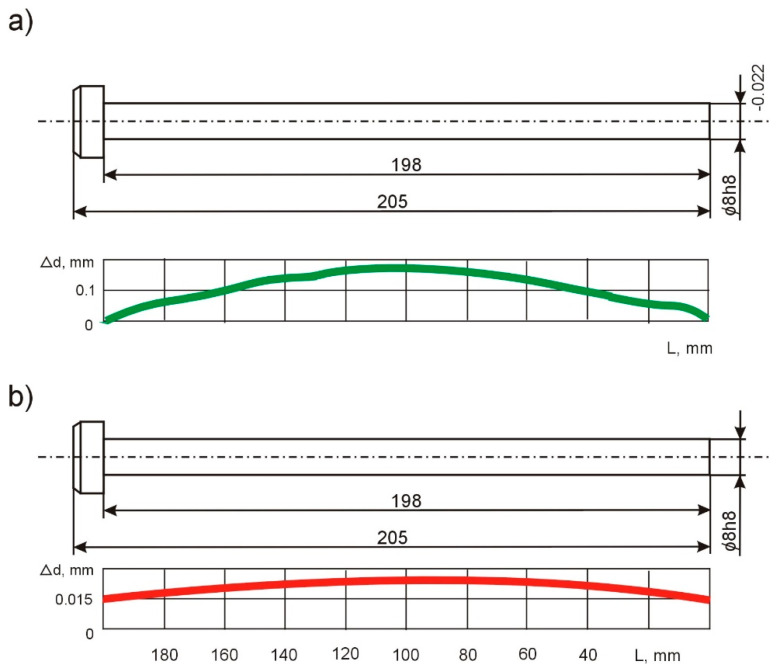
Longitudinal form error, Δ*d*, for single-pass shaft machining: (**a**) without the adapting device and (**b**) with the use of the device.

The initial experimental tests with the use of an adaptation device for machining shafts and axes of precision mechanics devices, *d* = 8 mm and *L* = 200 mm, show that it is possible to reduce the longitudinal shape inaccuracy by even seven times (Figure 10a,b).

## 4. Conclusions

As a part of the research work carried out, technological methods of machining shafts with low stiffness were developed, enabling the adjustment of the susceptibility of the technological system as a result of the design and application of a specially designed system for adjusting the susceptibility of the technological system during machining. In the control system of the compliance of the technological system, specially designed tailstocks were used, equipped with mechanisms enabling the regulation of the stiffness and software devices for controlling the machining.

The realized tests and experiments allowed us to draw the following conclusions:

- The geometric inaccuracy of the machine tool does not depend on the dimensions of the samples and the grinding conditions and can be reduced as a result of an increase in the stiffness and, thus, a reduction in the tailstock deflection.
- The susceptibility of the technological system can be compensated for by deliberately weakening the tailstocks (if there is no geometric inaccuracy in the machine tool, then the flexibility of the spindle and tailstock should be the same). The geometrical inaccuracy of the machine tool introduces a systematic inaccuracy to the machining’s results.
- In the case of the occurrence of geometrical inaccuracies of the machine tool, the technological system does not ensure an increase in the accuracy of the shape of the machined part.
- The uneven weakening of the centers enables the compensation of the shape deviation caused by the geometric inaccuracy of the machine tool and increases the machining’s accuracy.
- In comparison with the passive compliance equalization, the linearization control ensures a two-fold increase in the shape accuracy and in the software control, about a four times increase.
- Compared to the uncontrolled grinding process of shafts of low stiffness (with diameters *d* = 8 mm and *d* = 14 mm), the programmable compliance control increases the accuracy of the shape by one row.
• A further increase in the accuracy of the shape while automating the processes of the abrasive machining of the shafts with low stiffness is associated with the use of adaptive control.
• The machining of the shafts and axes of the precision mechanics adaptive devices (d = 8 mm and L = 200 mm) shows that it is possible to reduce the longitudinal shape inaccuracy by even seven times.

## 5. Patents

1. Lathe tailstock [patent no. 212961]/Victor Taranenko, Antoni Świć, Dariusz Wołos, Georgiy Taranenko—Official Gazette of the Patent Office, 2012, No. 12, p. 2897;
2. Lathe tailstock [patent no. 211537]/Victor Taranenko, Antoni Świć, Dariusz Wołos, Georgiy Taranenko—Official Gazette of the Patent Office, 2012, No. 5, p. 1073;
3. Lathe tailstock [patent no. 213606]/Victor Taranenko, Antoni Świć, Dariusz Wołos, Georgiy Taranenko, Jakub Szabelski—Official Gazette of the Patent Office, 2013, No. 4, p. 851;
4. Lathe tailstock [patent no. 214058]/Victor Taranenko, Antoni Świć, Dariusz Wołos, Georgiy Taranenko, Jakub Szabelski—Official Gazette of the Patent Office, 2013, No. 6, p. 1426.1.

## Figures and Tables

**Table 1:** Elastic flexibility and deformation of the tailstocks with a weakened section on the extension; *l_k_* = 70 mm.

Angle *α_k_* in (Degrees)	Cross-Sectional Area, *S* mm^2^	Moment of Inertia, mm^4^		Susceptibility, µm*/N*		Elastic Deformation, µm from Workforces	
		*I_y_*	*I_z_*	*F_p_*	*F_c_*	*F_P_* = 15 *N*	*F_c_* = 0.5 *F_P_*
				*ω_Y_* = 1.6 × 10^−3^·*l_k_*^3^/*I_Y_*	ω*_Z_* = 1.6 × 10^3^·*l_k_*^3^/*I_Z_*	*Y_Y_* = *F_P_*·*ω_Y_*	*Y_Z_ = F_c_*·*ω_Z_*
Tailstocks with a diameter of 32 mm							
120	147	610	5736	0.9	0.1	13.5	0.75
135	198	1233	8997	0.45	0.06	6.75	0.45
150	254	2326	13,037	0.24	0.04	3.6	0.3
180	378	6324	22,622	0.09	0.02	1.35	0.15
Tailstocks with a diameter of 22 mm							
120	74	231	1455	2.5	0.38	36.0	2.85
135	100	363	2282	1.5	0.24	22.5	1.8
150	128	659	3307	0.5	0.17	12.0	1.27
180	190	1604	5748	0.4	0.1	6.0	0.75

## Data Availability

Not applicable.
